# Acute kidney injury: Incidence, risk factors, and outcomes in severe COVID-19 patients

**DOI:** 10.1371/journal.pone.0251048

**Published:** 2021-05-25

**Authors:** Danilo Candido de Almeida, Maria do Carmo Pinho Franco, Davi Rettori Pardo dos Santos, Marina Colella Santos, Isabela Soucin Maltoni, Felipe Mascotte, Alexandra Aparecida de Souza, Paula Massaroni Pietrobom, Eduardo Alexandrino Medeiros, Paulo Roberto Abrão Ferreira, Flavia Ribeiro Machado, Miguel Angelo Goes

**Affiliations:** 1 Nephrology Division, Federal University of São Paulo, São Paulo, Brazil; 2 Laboratory of Applied Computing (LABCOM), Federal Institute of Education, Science and Technology of São Paulo, São Paulo, Brazil; 3 Infectious Disease Division, Federal University of São Paulo, São Paulo, Brazil; 4 Intensive Care Unit Division, Federal University of São Paulo, São Paulo, Brazil; Universidade de Sao Paulo Faculdade de Medicina, BRAZIL

## Abstract

**Background:**

COVID-19 is a multisystemic disorder that frequently causes acute kidney injury (AKI). However, the precise clinical and biochemical variables associated with AKI progression in patients with severe COVID-19 remain unclear.

**Methods:**

We performed a retrospective study on 278 hospitalized patients who were admitted to the ward and intensive care unit (ICU) with COVID-19 between March 2020 and June 2020, at the University Hospital, São Paulo, Brazil. Patients aged ≥ 18 years with COVID-19 confirmed on RT-PCR were included. AKI was defined according to the Kidney Disease Improving Global Outcomes (KDIGO) criteria. We evaluated the incidence of AKI, several clinical variables, medicines used, and outcomes in two sub-groups: COVID-19 patients with AKI (Cov-AKI), and COVID-19 patients without AKI (non-AKI). Univariate and multivariate analyses were performed.

**Results:**

First, an elevated incidence of AKI (71.2%) was identified, distributed across different stages of the KDIGO criteria. We further observed higher levels of creatinine, C-reactive protein (CRP), leukocytes, neutrophils, monocytes, and neutrophil-to-lymphocyte ratio (NLR) in the Cov-AKI group than in the non-AKI group, at hospital admission. On univariate analysis, Cov-AKI was associated with older age (>62 years), hypertension, CRP, MCV, leucocytes, neutrophils, NLR, combined hydroxychloroquine and azithromycin treatment, use of mechanical ventilation, and vasoactive drugs. Multivariate analysis showed that hypertension and the use of vasoactive drugs were independently associated with a risk of higher AKI in COVID-19 patients. Finally, we preferentially found an altered erythrocyte and leukocyte cellular profile in the Cov-AKI group compared to the non-AKI group, at hospital discharge.

**Conclusions:**

In our study, the development of AKI in patients with severe COVID-19 was related to inflammatory blood markers and therapy with hydroxychloroquine/azithromycin, with vasopressor requirement and hypertension considered potential risk factors. Thus, attention to the protocol, hypertension, and some blood markers may help assist doctors with decision-making for the management of COVID-19 patients with AKI.

## Introduction

The novel coronavirus disease (COVID-19) caused by the highly transmissible SARS-CoV-2 virus has shown diverse clinical manifestations and severe complications and has impacted health systems worldwide [1]. In March 2020, COVID-19 was declared a pandemic, and Brazil currently has one of the highest incidence and mortality rates [2]. Severe COVID-19 may represent a type of hyperimmune disorder and can frequently cause acute respiratory failure in critically ill patients [3]. In addition, this disease affects other organs, and there is growing evidence of kidney dysfunction in SARS-CoV-2-infected patients [4–6]. Although other mechanisms have been investigated [7], it is widely accepted that multiorgan involvement could be linked to the wide distribution of angiotensin-converting enzyme 2 receptor (ACE-2), which allows the SARS-CoV-2 virus to adhere to the host cell [6–9]. To date, ACE-2 expression has been found not only in the lungs, but also in the liver, stomach, ileum, colon, esophagus, and kidney [8].

In fact, the kidney distinctly expresses ACE-2 in its cells, including the proximal tubule cells, mesangial cells, parietal epithelium of the Bowman’s capsule, collecting ducts, and podocytes [8–13]. Based on this evidence, several studies have already identified SARS-CoV-2 in the kidney during post-mortem autopsies of COVID-19 patients, suggesting that kidney tropism can be associated with acute kidney injury (AKI) [13–17]. In this context, other studies have highlighted the prevalence of kidney disease on admission and reported a high prevalence of AKI during hospitalization in patients with COVID-19, which was also associated with in-hospital mortality [18–21].

AKI differs according to COVID-19 severity. In mild to moderate cases, AKI is not common and is determined by subclinical kidney abnormalities [12–18]. In contrast, AKI is common among critically ill patients with COVID-19, affecting approximately 20%-40% of patients admitted to intensive care units (ICUs) [13, 19–22]. Possible causes of AKI in severe or critical COVID-19 patients include volume depletion, inflammation and hemodynamic changes, viral infection-associated tubular injury, thrombotic vascular processes, glomerular pathologies, and rhabdomyolysis [23]. With respect to severe COVID-19 cases, other reports have described additional risk factors associated with AKI such as respiratory failure, need for mechanical ventilation, older age, diabetes mellitus, cardiovascular disease, and Black race [1, 14, 21]. Moreover, patients with COVID-19-associated AKI were more likely to require kidney replacement therapy than those without COVID-19 [18, 19, 21].

Although there are recent findings showing an association between AKI and COVID-19, the precise relationship of clinical and biochemical variables with COVID-19-associated AKI remains unclear. Hence, in the present study, we aimed to describe the incidence, risk factors, and outcomes of AKI in patients with severe COVID-19.

## Materials and methods

### Study design and measurements

We performed a retrospective study on a subset of hospitalized patients with severe and critical COVID-19, who were admitted between March and June 2020 at Hospital São Paulo (Federal University of São Paulo), in São Paulo, Brazil. The inclusion criteria were: age ≥ 18 years, and confirmed SARS-CoV-2 infection by RT-PCR. The exclusion criteria were pregnancy, stage 5 chronic kidney disease under dialysis, transplanted kidney, human immunodeficiency virus infection, hepatitis B and C, use of immunosuppressants, and malignancies. Obesity was defined as a body mass index ≥ 30 kg/m^2^ [24]. Oliguria was defined as diuresis of ≤ 400 ml/24 h. AKI was defined as an increase in serum creatinine of 0.3 mg/dl in 48 h or by 1.5 times in 7 days, or as diuresis lower than < 0.5 mL/kg/hour during the 6 hours from hospital admission. AKI was defined and stratified according to the Kidney Disease Improving Global Outcomes (KDIGO) criteria [25]. We performed the KDIGO criteria two different times (within 48 h, and 7 days after hospital admission). Thereafter, we classified the patients into two groups according to kidney function status: with AKI (CoV-AKI), and without AKI (non-AKI).

All demographic and clinical data, including blood cell counts, blood gas analysis, biochemical variables, urine output, medications, mechanical ventilation requirement, comorbidities (smoking, diabetes, hypertension, obesity, and respiratory, cardiac, or hepatic diseases), need for kidney replacement therapy, and complications were obtained through electronic medical records. The estimated glomerular filtration rate (eGFR) was calculated using the CKD Epidemiology Collaboration formula during early hospitalization and after 7 days of ward or ICU stay; thus, we utilized creatinine levels at the peak of hospitalization in each period [26]. Acute respiratory distress requiring mechanical ventilation was defined as acute onset hypoxemia with bilateral pulmonary opacities on chest tomography and PaO_2_/FIO_2_ ≤ 300 [27].

All COVID-19 patients were treated according to the standard protocols for both wards and ICUs. Hemodynamically stable CoV-AKI patients who required kidney replacement therapy underwent standard intermittent hemodialysis, while all critically ill hemodynamically unstable patients underwent prolonged intermittent kidney replacement therapy. Vasoactive agents (usually norepinephrine) were used when mean arterial pressure persistently presented as <65 mmHg in patients with adequate intravascular fluid status [28]. All routine tests and patient management were at the discretion of the attending physician, and our research team did not interfere with any aspects of diagnosis or treatment.

The study was approved by the local ethics committee on human research at Hospital São Paulo, UNIFESP, Brazil (no. 0555/2020), and informed consent was waived due to the observational nature of the study. The study team analyzed only the anonymized data.

### Statistical analysis

Categorical variables are presented as frequencies and percentage distributions. All continuous variables were examined for normality using the Shapiro-Wilk test, and analyzed using the Mann-Whitney test. Data are expressed as median, interquartile range, and minimum-maximum values. The analyses were performed by stratifying patients according to the presence or absence of AKI. Logistic regression analyses were performed to establish the degree of association between various risk factors and AKI after COVID-19 infection. For this procedure, the cut-off points (>50th percentile) were adopted for some variables of the biochemical profile (i.e., C-reactive protein, D-dimer) and complete blood count. Multivariate logistic regression models were fitted to determine the presence of confounding factors. All variables showing a p<0.20 in the univariate analysis were presented to a single multivariate model. Variables were retained in the model if a Wald test revealed p<0.05. Data are expressed as odds ratios (ORs) and 95% confidence intervals (CIs). Statistical analyses were performed using SPSS version 22 (IBM, Armonk, New York, USA), and images were obtained using GraphPad Prism (version 8.0; La Jolla, CA, USA).

## Results

**Fig 1:**
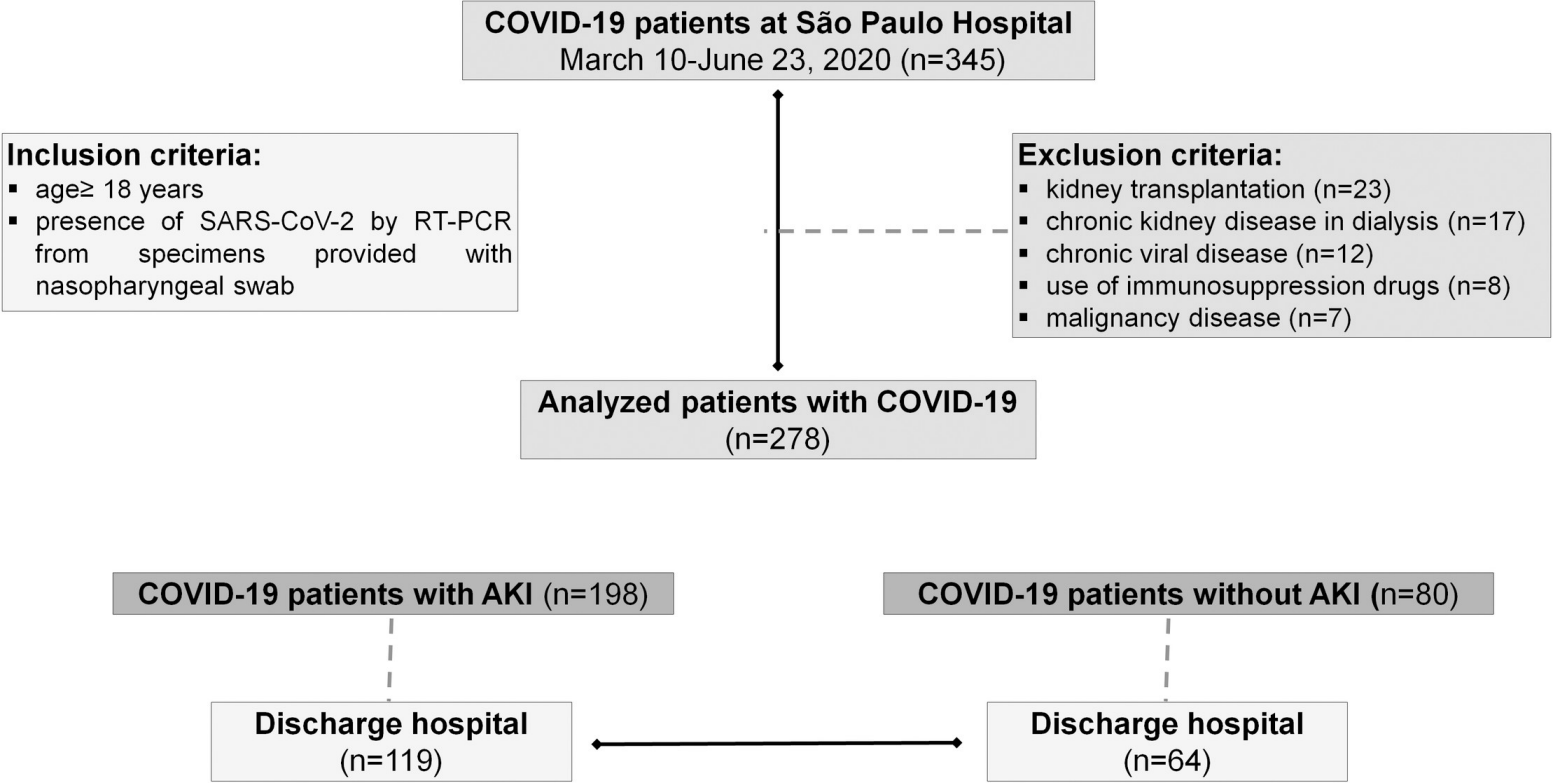
Workflow diagram of the patient selection. A total of 345 patients SARS-CoV-2 confirmed by RT-PCR from specimens provided with nasopharyngeal swab were selected from Hospital São Paulo of Federal University of São Paulo. After exclusion criteria, the remaining patients (n = 278) were subdivided into two subgroups: 1) COVID-19 patients with AKI (n = 198) and 2) COVID-19 patients without AKI (n = 80).

During the study period, 345 patients were admitted, and after 67 individuals were excluded. Then, 278 severe and critically ill hospitalized COVID-19 patients were enrolled (185 women and 93 men), aged around 60 years (range: 18–97 years) (Fig 1). Of these patients, 60.4% were admitted to ICUs; however, all patients were managed in ICUs after 3–5 days of hospitalization. The total hospitalization time ranged from 1 day to 27 days, and overall hospital mortality was approximately 34.17% (n = 95).

According to the KDIGO criteria for staging of AKI, we observed that: i) 72 patients (25.9%) had KDIGO 1, ii) 25 patients (8.9%) had KDIGO 2, and iii) 10 patients had KDIGO 3 (3.6%), within 48 hours of hospital admission. Consequently, after 5–7 days, most COVID-19 patients had acute kidney injury (AKI) during hospitalization (n = 198; 71.2%). The KDIGO criteria for AKI staging were also assessed after 7 days of ICU stay and we found 52 patients (26.3%) in KDIGO 1, 25 patients (12.6%) in KDIGO 2, and 121 patients (61.1%) in KDIGO 3. Thus, we classified all COVID-19 patients into two groups: those with AKI, designated as CoV-AKI (n = 198); and those without AKI, denoted as non-AKI (n = 80) (Fig 1).

In general, to identify the classificatory tendencies in the AKI group, we explored unsupervised learning linear algorithms to promote reduction of dimensionality between the several clinical parameters investigated. We then used principal component analysis (PCA), and did not identify any cluster evidence for both the CoV-AKI and non-AKI groups (S1a Fig in S1 File). Afterwards, the clustering heat map did not demonstrate any grouping propensities; however, when the vectorial contribution of variables used was analyzed, an important participation of white series in the blood test and some arterial gasometric parameters were also confirmed by squared cosine analysis of individual PCA components (S1b–S1d Fig in S1 File).

In sequence, our global comparative investigation showed that age, gender, and race were similar between the groups analyzed (Table 1). However, CoV-AKI patients presented with higher levels of inflammatory and kidney markers at admission (CRP and sCr). Furthermore, as shown on vectorial analysis of PCAs, CoV-AKI patients had increased indexes of leucocytes, neutrophils, monocytes, and neutrophils-to-leucocytes ratio (NLR), in comparison with the non-AKI group. The red series of blood tests and arterial blood gas values were not significantly altered (Table 1). Moreover, we observed oliguria (23.1%) and a need for kidney replacement therapy (47.5%) after 1.5 days (range: 1–7 days) of hospitalization in the CoV-AKI group.

**Table 1:** Demographic and biochemical data at hospital admission.

Variables	non-AKI group	CoV-AKI group	*p Value*
	(n = 80)	(n = 198)	
Age (years)	58.0 (17.5)	59.8 (20.0)	0.079
Gender (%)			
Male	36.3	32.3	0.530
Female	63.8	67.7	
Race (%)			
White	52.6	59.5	
Black	10.3	11.6	0.419
Brown	37.2	28.9	
sCr (mg/dL)	0.75 (0.30)	0.82 (0.50)	0.009*
CRP (mg/L)	119.0 (194.0)	208.0 (220.1)	0.001*
D-dimer (μg/L)	1.62 (2.1)	1.82 (2.9)	0.310
*Red Cell Profile/Indices*			
Hemoglobin (g/dL)	13.6 (2.8)	13.5 (3.1)	0.107
Hematocrit (%)	40.3 (6.9)	39.7 (9.0)	0.098
MCV (fL)	88.9 (7.5)	90.7 (7.1)	0.067
MCH (pg)	30.1 (3.0)	30.6 (2.4)	0.054
MCHC(g/dL)	33.7 (1.7)	33.8 (1.4)	0.985
RDW (%)	13.6 (1.8)	13.6 (1.7)	0.607
*White Blood Cell Profile*			
Leukocyte (cell/μL)	7780 (4810)	9280 (5490)	0.010*
Neutrophil (cell/μL)	5710 (4301)	7138 (4451)	0.001*
Eosinophil	0 (41)	0 (35.5)	0.423
Basophil (cell/μL)	13 (23)	8 (20)	0.715
Lymphocyte (cell/μL)	1200 (756)	1022 (788)	0.596
Monocyte (cell/μL)	433 (390)	475 (378)	0.021*
Platelet (x10^6^cell/μL)	1.9 (1.0)	1.9 (9.2)	0.622
NLR	5.2 (4.6)	7.2 (6.9)	0.022*
PLR	175.9 (134.1)	175.7 (158.5)	0.924
*Acid-Base Profile*			
pH	7.45 (0.08)	7.44 (0.09)	0.592
pCO_2_ (mmHg)	34.4 (9.5)	32.5 (8.9)	0.191
HCO_3_ (mEq/L)	21.5 (4.5)	21.1 (5.1)	0.060
Anion-Gap (mEq/L)	7.7 (4.4)	8.6 (4.8)	0.213
PaO_2_/FiO_2_ (mmHg)	237.0 (140.3)	230.0 (142.5)	0.336

Overall variables description between COVID-19 AKI patients and COVID-19 non-AKI patients. sCr: Serum Creatinine; CRP: C-reactive protein; MCV: Mean Corpuscular Volume; MCH: Mean Corpuscular Hemoglobin; MCHC: Mean Corpuscular Hemoglobin Concentration; RDW: Red Cell Distribution Width; NLR: Neutrophil-to-Lymphocyte Ratio; PLR: Platelet-to- Lymphocyte Ratio; PCO_2_: Partial Pressure of Carbon Dioxide; HCO_3_: Bicarbonate; PaO_2_/FiO_2_: Arterial Oxygen Partial Pressure to Fractional Inspired Oxygen Ratio. Data were expressed as median (Interquartile Range) and analyzed using Mann-Whitney test (*p<0.*05*).

**Fig 2:**
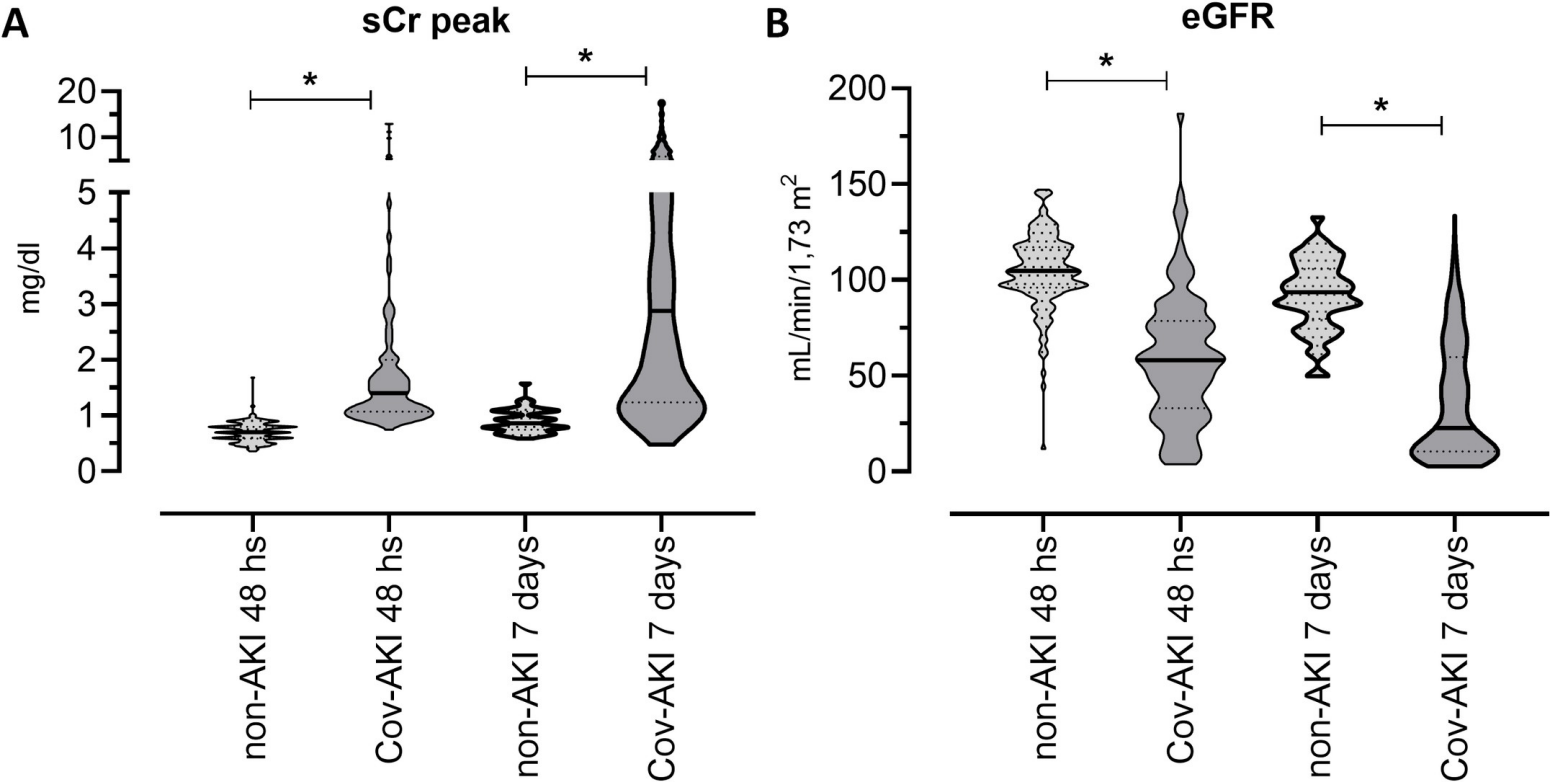
Evaluation of kidney function during hospitalization. (A) serum creatinine levels at admission and median peak creatinine after 7 days of hospitalization in COVID-19 patients with AKI (CoV-AKI) and without AKI (non-AKI); and (B) median estimate of the glomerular filtration rate (eGFR) at admission and after 7 days of hospitalization in COVID-19 patients with AKI (CoV-AKI) and without AKI (non-AKI) (* p <0.05).

In addition, we observed higher levels of early serum creatinine and lower eGFR within 48 hours of hospitalization in the CoV-AKI group than in the non-AKI group. The same behavior was observed after seven days of hospitalization (Fig 2). We found no difference in hematuria between the CoV-AKI and non-AKI groups. We found that most CoV-AKI patients were hospitalized in the ICU (Table 2), and not surprisingly, the mortality rate was higher in this same group, with an approximately 2-fold increase to that in patients without AKI (39.8% vs. 20%, p = 0.002; data not shown). We observed that the mortality rate was further elevated in the CoV-AKI group stratified according to the KDIGO 3 criteria (64.9%; p<0.001; data not shown).

**Table 2:** Overall description of comorbidities, medication usage and general complications.

Variables	non-AKI group	CoV-AKI group	*p Value*
	(n = 80)	(n = 198)	
Hospitalization ICU (%)	30 (37.5%)	138 (69.7%)	<0.001*
***Comorbidities (%)***			
Hypertension			
No	46.1	29.5	0.010*
Yes	53.9	70.5	
Type 2 Diabetes			
No	67.1	58.4	0.190
Yes	32.9	41.6	
Smoking			
No	76.3	66.8	0.129
Yes	23.7	33.2	
Obesity			
No	85.5	83.2	0.635
Yes	14.5	16.8	
Chronic Respiratory Disease			
No	85.5	83.7	0.758
Yes	14.5	16.3	
Cardiopathy			
No	81.6	77.9	0.506
Yes	18.4	22.1	
Hepatic Diseases			
No	98.7	93.6	0.085
Yes	1.3	6.4	
Previous AKI			
No	92.1	85.3	0.135
Yes	7.9	14.7	
*Medication Use (%)*			
Hydroxychloroquine			
No	93.8	98.0	0.071
Yes	6.2	2.0	
Azithromycin			
No	73.8	78.3	0.416
Yes	26.2	21.7	
Hydroxycloroquine+Azithromycin			
No	68.8	49.5	0.003*
Yes	31.2	50.5	
Vasopressor agents			
No	93.7	50.9	<0.001*
Yes	6.3	49.1	
*Complications (%)*			
Sepsis			
No	38.8	45.5	0.759
Yes	61.3	54.5	
SOFA Score			
0–6	83.7	65.4	
7–9	8.2	18.7	0.123
10–12	8.2	9.3	
13–14	0	5.6	
15	0	0.9	
15–24	0	0	
Mechanical Ventilation			
No	85.4	44.5	< 0.001*
Yes	14.6	55.5	

Description of comorbidities, medication usage and general complications between COVID-19 AKI patients and COVID-19 non-AKI patients. SOFA: Sequential Organ Failure Assessment. Data were expressed as percentage and analyzed using Chi-Square test. (*p<0.05).

In addition, we did not find differences in the frequencies of diabetes, smoking, obesity, chronic respiratory diseases, cardiopathy, hepatic diseases, sepsis incidence, SOFA (sequential organ failure assessment score) score, acid-base parameters, and use of renin-angiotensin system blockers, between the groups investigated. Interestingly, CoV-AKI patients were more hypertensive (70.5% vs. 53.9%) and made more use of combined therapy comprising hydroxychloroquine and azithromycin (50.5% vs. 31.2%). In addition, the CoV-AKI group required more vasopressor drugs (49.1% vs. 6.3%) and mechanical ventilation (55.5% vs. 14.6%) than the non-AKI group.

Univariate logistic analysis revealed that AKI in COVID-19 patients was associated with older age, history of hypertension, mechanical ventilation, vasoactive drugs, combined treatment with hydroxychloroquine and azithromycin, and higher basal levels of CRP, MCV, leukocytes, neutrophils, and NLR (Table 3). Moreover, we performed a progressive multivariate logistic regression model fitted with all covariates that tended to correlate with AKI development. Hypertension and the use of vasoactive drugs were independent risk factors associated with AKI in COVID-19 patients (Table 3).

**Table 3:** Logistic regression analyses of factors associated with AKI in COVID-19-positive patients.

	Univariate Logistic Model		Multivariate Logistic Model	
	OR (95% CI)	*P Value*	OR (95% CI)	*P Value*
Male Sex (no/yes)	1.91 (0.69–2.05)	0.530		
Age (>62 years)	1.68 (0.99–2.83)	0.050*		
Hypertension (no/yes)	2.04 (1.18–3.54)	0.001*	2.62 (1.21–5.67)	0.015
Type 2 Diabetes (no/yes)	1.45 (0.83–2.53)	0.191		
Smoking (no/yes)	1.59 (0.87–2.94)	0.131		
Obesity (no/yes)	1.20 (0.57–2.52)	0.636		
Respiratory Disease (no/yes)	1.16 (0.55–2.45)	0.698		
Cardiopathy (no/yes)	1.26 (0.64–2.47)	0.506		
Hepatic Diseases (no/yes)	5.11 (0.65–40.0)	0.220		
Previous AKI (no/yes)	2.00 (0.79–5.06)	0.141		
CRP (>187.5 mg/L)	2.44 (1.41–4.22)	0.001*		
D-dimer (> 1.77 μg/L)	1.21 (0.69–2.14)	0.502		
Hemoglobin (>13.5 g/dL)	0.94 (0.56–1.58)	0.822		
Hematocrit (> 39.7%)	0.76 (0.45–1.29)	0.308		
MCV (> 90.3 fL)	1.80 (1.06–3.05)	0.029*		
MCH (> 30.5 pg)	1.59 (0.94–2.69)	0.082		
RDW (> 13.6%)	0.92 (0.55–1.55)	0.763		
MCHC (> 33.8 g/dL)	1.27 (0.75–2.13)	0.377		
Leukocyte (> 8590 cell/μL)	2.16 (1.26–3.70)	0.005*		
Neutrophil (> 6662 cell/μL)	2.22 (1.30–3.80)	0.003*		
Eosinophil (> 0 cell/μL)	1.19 (0.70–1.99)	0.522		
Basophil (> 10 cell/μL)	0.92 (0.55–1.55)	0.763		
Lymphocyte (> 1048 cell/μL)	0.76 (0.45–1.27)	0.290		
Monocyte (> 466 cell/μL)	1.25 (0.74–2.10)	0.405		
Platelet (> 1.9x10^6^/μL)	1.07 (0.64–1.80)	0.791		
NLR (> 6.4)	2.24 (1.31–3.83)	0.003*		
PLR (> 175.9)	1.02 (0.60–1.68)	0.989		
Hydroxychloroquine use (no/yes)	3.23 (0.85–12.37)	0.286		
Azithromycin use (no/yes)	0.78 (0.43–1.43)	0.417		
Hydroxychloroquine+Azithromycin use (no/yes)	2.25 (1.29–3.89)	0.004*		
Mechanical Ventilation (no/yes)	7.29 (3.01–17.67)	0.001*		
Vasoactive Drug (no/yes)	14.46 (4.24–49.34)	<0.001	14.36 (4.14–49.8)	<0.001

*Data are reported as odds ratio (OR) and 95% Confidence Interval (95% CI)*. *All variables showing a p<0*.*20 in the univariate analysis were presented to the multivariate models by using the forward method*. *Variables were retained in the model if a Wald test revealed*.

**Fig 3:**
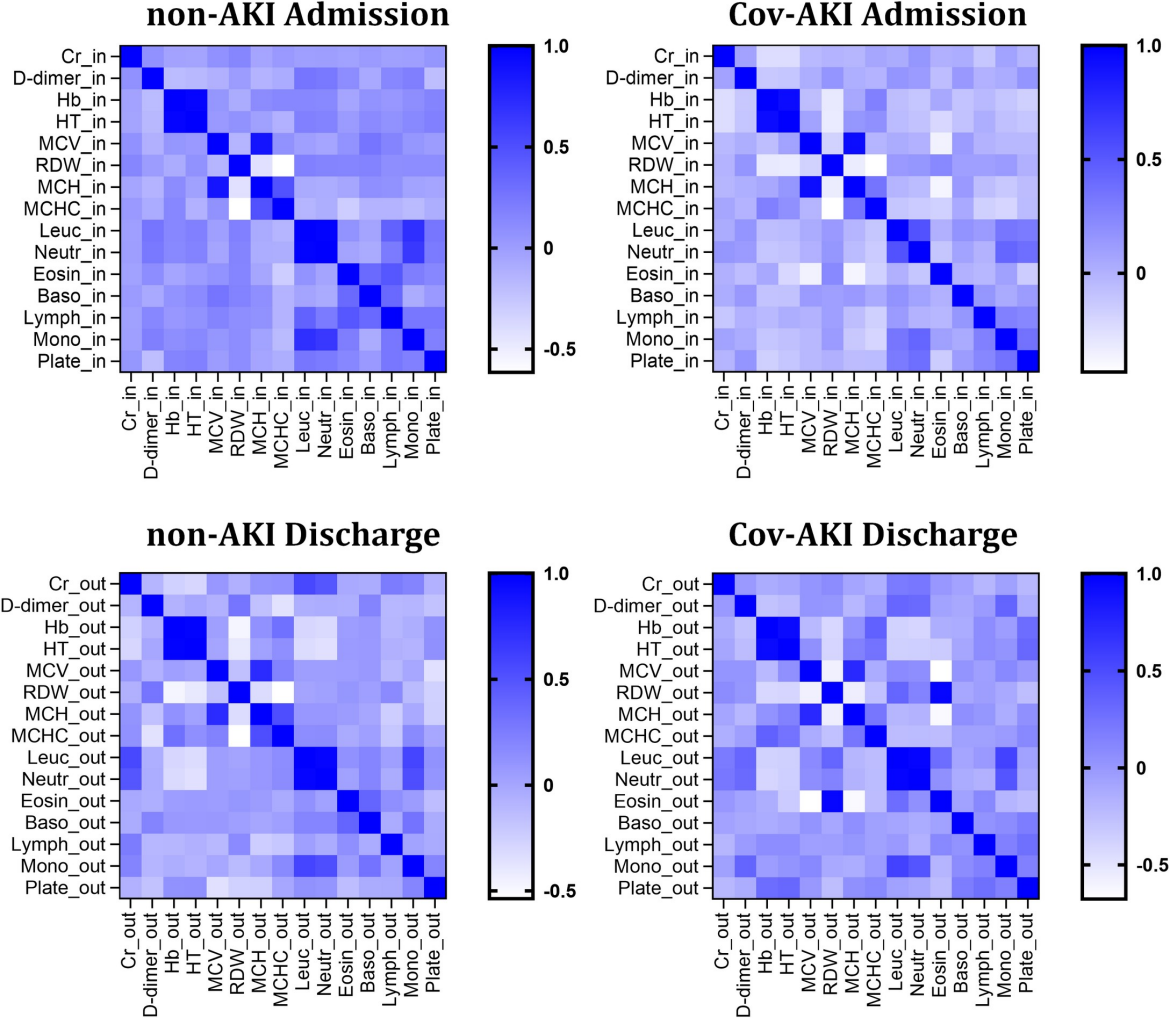
Correlation matrix among clinical variables at hospital admission and discharge. COVID-19 patients with AKI (Cov-AKI) and without AKI (non-AKI) were analyzed by Euclidean Distance Matrix according to relevant serological and blood markers between the admission and discharge periods of hospitalization. 2-tailed Pearson Correlation was ranged from 1 (blue color) to -1 (white color); (data with p<0.05).

To investigate whether biochemical and blood-associated parameters could help physicians in decision-making for managing patients with COVID-19 and AKI, we carried out a multi-correlation matrix analysis and identified different profiles between groups (CoV-AKI and non-AKI) at admission and discharge. With consideration to only pronounced differences, we detected a positive correlation between monocytes and both leucocytes and neutrophils in the non-AKI group at admission. In contrast, at admission, there was a negative correlation between RDW and both hemoglobin and hematocrit, and between eosinophils with MCV and MCH (Fig 3), in CoV-AKI patients.

We observed a positive correlation of creatinine with both leukocytes and neutrophils during hospital discharge in the non-AKI group. Moreover, there was a negative correlation between RDW and anemia parameters (hemoglobin, hematocrit, MCH, and MCHC). On the other hand, in CoV-AKI patients at discharge, we found an exclusive and intriguing relationship between eosinophils and anemia markers. Eosinophils were positively correlated with RDW and negatively correlated with MCV and MCH (Fig 3). Finally, using direct group-group comparisons, our findings at hospital discharge indicated that CoV-AKI patients presented an altered red/white hematological profile (hemoglobin, hematocrit, and MCHC; and leukocyte, neutrophil, monocyte, and NLR) with elevated inflammatory markers (D-dimer and RDW), when compared to the non-AKI group (Table 4). Altogether, these results suggest an association between hematological and inflammatory parameters on blood tests at admission that could help in the interpretation and management of AKI in COVID-19 patients.

**Table 4:** Description of biochemical data and blood cellular profile at hospital discharge.

Variables	non-AKI group	CoV-AKI group	*p Value*
	(n = 64)	(n = 119)	
sCr (mg/dL)	0.96 (0.23)	1.43 (0.47)	0.087
D-dimer (μg/L)	1.90 (2.8)	2.57 (4.1)	0.080
*Red Cell Profile/Indices*			
Hemoglobin (g/dL)	13.2 (2.8)	10.6 (3.8)	0.001*
Hematocrit (%)	38.9 (6.6)	30.2 (10.9)	0.001*
MCV (fL)	89.2 (7.2)	91.9 (4.4)	0.024*
MCH (pg)	30.2 (3.5)	30.3 (2.2)	0.469
MCHC (g/dL)	34.0 (1.3)	31.9 (1.7)	0.010*
RDW (%)	13.6 (1.6)	16.1 (4.1)	0.002*
*White Blood Cell Profile*			
Leukocyte (cell/μL)	6470 (3260)	7940 (3640)	0.002*
Neutrophil (cell/μL)	4086 (2393)	4902 (2961)	0.003*
Eosinophil (cell/μL)	172 (180.5)	175 (197)	0.851
Basophil (cell/μL)	25 (42)	33 (43)	0.402
Lymphocyte (cell/μL)	1134 (756)	1009 (788)	0.317
Monocyte (cell/μL)	498 (311)	607 (359)	0.028*
Platelet (x10^6^cell/μL)	3.1 (1.8)	3.0 (1.5)	0.939
NLR	2.3 (2.0)	3.2 (3.1)	0.011*
PLR	181.5 (123.2)	156.2 (98.3)	0.601

General description of biochemical data at hospital discharge for all patients. MCV: Mean Corpuscular Volume; MCH: Mean Corpuscular Hemoglobin; MCHC: Mean Corpuscular Hemoglobin Concentration. RDW: Red Cell Distribution Width; NLR: Neutrophil-to-Lymphocyte Ratio; PLR: Platelet-to-Lymphocyte Ratio. Data were expressed as median (Interquartile Range). Data were analyzed using Mann-Whitney test.

(*p<0.05).

## Discussion

In this single-center retrospective observational cohort study, we found that hematological and inflammatory markers, hypertension, mechanical ventilation, and therapy with hydroxychloroquine/azithromycin or vasopressors were associated with AKI in patients with severe COVID-19.

Since the onset of the COVID-19 pandemic, several studies have focused on multiple organ dysfunction syndrome [1, 22], but none have investigated associated blood parameters, technical procedures, or medicines. First, we observed an elevated AKI incidence (>70%), with more than half of these patients falling under the KDIGO 3 criteria within 7 days of hospitalization. This frequency was higher than that seen in other studies [23, 29, 30] however, our population was represented by critically ill COVID-19 patients with relevant comorbidities or dysfunction. Most AKI patients showed elevated levels of kidney creatinine (sCr) and inflammatory markers (C-reactive protein, leukocytes, neutrophils, monocytes, and NLR) at admission. During viral infection, white blood cells fight against the offending agent by producing cytokines that stimulate the liver to produce C-reactive protein. This inflammatory process is a risk factor for AKI, and other reports have identified C-reactive protein levels and altered white blood cell count as predictors of COVID-19 severity in patients with kidney function impairment [31, 32]. Indeed, CoV-AKI patients in our study had higher sCr and reduced eGFR than those in the non-AKI group during the hospitalization period. Furthermore, we also found that C-reactive protein, leukocytes, neutrophils, and NLR were associated with AKI in COVID-19 patients, corroborating those findings reported by other studies [20, 29]. Although bordered by controversies [33] these results demonstrate a possible relationship between exacerbated inflammation and AKI development in COVID-19 patients [34].

Curiously, our study is the first to show that combined hydroxychloroquine and azithromycin treatment is associated with kidney dysfunction in COVID-19 patients. Although there are different combinations, and associations with other parameters in several contexts [35, 36] (for instance, the fact that severe patients made more use of this regimen), we believe that a causal relationship may exist between this combined treatment and AKI. In fact, Gevers et al. reported that COVID-19 patients treated with hydroxychloroquine may be more susceptible to its adverse effects, in part due to a multiple organ dysfunction secondary to SARS-CoV-2 infection [37]. Furthermore, hydroxychloroquine could potentially induce or intensify AKI by increasing lysosomal pH and inhibiting autophagy [38]. It has been demonstrated that chloroquine inhibits autophagic flux by impairing autophagosome-lysosome fusion [38]. Moreover, hydroxychloroquine can induce disorganization of the Golgi and endosomal-lysosomal systems, thereby increasing oxidative stress. Considering this evidence and that autophagy seems to be important for tubular proliferation and repair, chloroquine treatment can affect tubular cell metabolism and kidney function [38, 39]. Since hydroxychloroquine is cleared by the kidney and liver, severely ill patients may thus have adverse reactions [36, 37].

In line with the attention to combined treatment, Wong et al. reported a case of an episode of hypotension after infusion of azithromycin [40]. Additionally, in another case report, Woodruff et al. reported the development of acute interstitial nephritis requiring hemodialysis, following azithromycin treatment [41]. Thus, it is plausible to suspect that azithromycin associated with hydroxychloroquine could be detrimental to kidney function by its cellular toxicity, and that the immune and cardiovascular alterations caused can induce hemodynamic changes and consequently, low kidney perfusion, aggravating AKI [35–42].

Indeed, our findings highlight a critical need to better understand disease-and treatment-specific factors that drive the risk of AKI in COVID-19 patients, considering that acute tubular injury, the most predominant manifestation, was detected in the postmortem biopsy of infected individuals [5, 6, 12, 13, 18]. In this case, the pathophysiology of AKI in COVID-19 was not completely understood. To date, some researchers have demonstrated that AKI in COVID-19 patients was related to acute tubular injury derived from ischemic or toxic injury [1, 4, 5, 7].

Acute tubular injury may occur in the setting of prolonged volume depletion, at hemodynamic states that reduce kidney perfusion, and by toxic effects resulting from medicines on kidney tubular cells [3–6, 13]. Severe SARS-CoV-2 infection promotes immune cell recruitment and intense cytokine release, leading to hemodynamic instability and low kidney perfusion [3, 5, 14, 17]. However, we cannot discard that AKI may also result from other multifactorial causes. For instance, components of dysregulated inflammation, ischemia-reperfusion injury, coagulation, and endothelial cell dysfunction, all of which are improved by viral invasion, can directly damage kidney cells [1, 4–7, 17, 18].

After adjusting for confounders in our final multivariate model, we also detected hypertension and the use of vasoactive drugs as potential predictors and risk factors independently associated with AKI in COVID-19 patients. Some comorbidities were already associated with AKI in severe SARS-CoV-2 infection in other studies [20, 21, 23, 29, 36]. The association between hypertension and AKI in COVID-19 patients may be partially explained by the chronic and subclinical changes in kidney function promoted by the utilization of some medicines as diuretics, thus decreasing its interest as a potential predictor [43]. However, although only identified in the univariate model, neutrophils could be considered excellent predictive candidates. They have already been reported as a risk factor with the association of NLR with stage 2 or 3 AKI in hospitalized individuals with COVID-19. They were also used to predict critical evolution at early stages [20, 44]. In this context, patients with severe COVID-19 can be considered as presenting a novel form of viral sepsis, with progressive lymphopenia associated with neutrophilia and monocytosis. These patients are characterized by systemic hyperinflammation, coagulopathy, and organ damage [43–46].

Therefore, this inflammatory milieu can cause hemodynamic instability with low tissue perfusion, and require mechanical ventilation and vasoactive amines. In fact, other reports have shown that ventilation support in acute respiratory failure was independently associated with AKI in COVID-19 patients, corroborating our findings in the univariate models [28, 43–48]. Moreover, we identified that requiring vasoactive drugs, such as norepinephrine, was both a risk factor and predictor (>14-fold increase) of AKI in COVID-19 patients. This evidence indicates that patients who required vasoactive drugs and mechanical ventilation had more severe acute kidney injury. Similarly, Chou et al. observed that the use of vasopressor agents in critically ill patients undergoing kidney replacement therapy was associated with worse outcomes [49]. Other researchers found that norepinephrine infusion affected intrarenal oxygenation, contributing to sepsis-associated AKI in an ovine experimental model [50].

Furthermore, in our study, patients with CoV-AKI presented hematological alterations, resulting in an anemic profile during hospital discharge. Anemia commonly causes deterioration of respiratory diseases and is associated with the severity of COVID-19. Tao et al. reported that anemia was an independent risk factor associated with severe COVID-19 [51]. In our findings, anemia could be associated with the severity of CoV-AKI, since anemia occurs in critically ill patients mainly due to blood loss caused by several tests, dialysis, invasive procedures, inflammation, deficiency in erythropoietin production, decreased retention of uremic solutes, and kidney dysfunction [52–55].

Finally, we observed some modulation in the profile of certain clinical variables that were correlated during admission and discharge in both the groups analyzed. We verified a severe inflammatory pattern in all COVID-19 patients at admission. However, we also exclusively detected the presence of an inverse relationship between eosinophils and MCV (also identified in the univariate model), Hb, and hematocrit in the CoV-AKI patients, which was not seen in non-AKI patients and persisted at hospital discharge. Moreover, RDW and eosinophils were only strongly positively correlated at discharge in patients with kidney impairment. Interestingly, these relationships can facilitate AKI interpretation and management at admission in COVID-19 patients and are useful for assisting doctors with decision-making during treatment. Considering our findings, inflammatory mediators could play an important role in anemia during hospital discharge by inferring hemodynamic involvement during the disease course. Thus, uncontrolled inflammation may promote structural and functional modifications in red blood cells, which may affect their deformability, reducing tissue microvascular perfusion, and promoting multiple organ failure [44].

Our present study has some potential limitations. First, this was a retrospective cohort study with a small number of patients. Second, the study was conducted in a single center, and there was no intervention by the researchers, sequential analysis, or decision-making regarding the outcomes and requirement for hemodialysis maintenance. Lastly, given the large number of potential predictors evaluated and the initial lack of selection, as guided by the variable hypotheses, we cannot rule out the possibility of confounding bias. Despite these limitations, a major strength of the current study is that our observation serves as a warning about the use of hydroxychloroquine along with azithromycin in AKI patients with COVID-19.

In conclusion, our study involving critically ill hospitalized patients with COVID-19 documented an interesting relationship shown by AKI evolution with hemodynamic instability, inflammation, and the combined use of hydroxychloroquine and azithromycin. Thus, attention to protocols should be considered crucial for COVID-19 patients with AKI, and inflammatory and hematological markers on blood tests at admission may help facilitate decision-making for treatment interventions.

## Supporting information

S1 File (PDF)

## References

[1] Huang C, Wang Y, Li X, Ren L, Zhao J, Hu Y, et al. Clinical features of patients infected with 2019 novel coronavirus in Wuhan, China. Lancet. 2020;395(10223):497–506. 10.1016/S0140-6736(20)30183-5 31986264 PMC7159299

[2] Organization WH. Coronavirus Disease (COVID-19) Dashboard 2020 [cited 2002 november]. Available from: https://covid19.who.int/region/amro/country/br

[3] McGonagle D, Sharif K, O’Regan A, Bridgewood C. The Role of Cytokines including Interleukin-6 in COVID-19 induced Pneumonia and Macrophage Activation Syndrome-Like Disease. Autoimmun Rev. 2020;19(6):102537. 10.1016/j.autrev.2020.102537 32251717 PMC7195002

[4] Su H, Yang M, Wan C, Yi LX, Tang F, Zhu HY, et al. Renal histopathological analysis of 26 postmortem findings of patients with COVID-19 in China. Kidney Int. 2020;98(1):219–27. 10.1016/j.kint.2020.04.003 32327202 PMC7194105

[5] Pei G, Zhang Z, Peng J, Liu L, Zhang C, Yu C, et al. Renal Involvement and Early Prognosis in Patients with COVID-19 Pneumonia. J Am Soc Nephrol. 2020;31(6):1157–65. 10.1681/ASN.2020030276 32345702 PMC7269350

[6] Naicker S, Yang CW, Hwang SJ, Liu BC, Chen JH, Jha V. The Novel Coronavirus 2019 epidemic and kidneys. Kidney Int. 2020;97(5):824–8. 10.1016/j.kint.2020.03.001 32204907 PMC7133222

[7] Harrison AG, Lin T, Wang P. Mechanisms of SARS-CoV-2 Transmission and Pathogenesis. Trends Immunol. 2020;41(12):1100–15. 10.1016/j.it.2020.10.004 33132005 PMC7556779

[8] Zou X, Chen K, Zou J, Han P, Hao J, Han Z. Single-cell RNA-seq data analysis on the receptor ACE2 expression reveals the potential risk of different human organs vulnerable to 2019-nCoV infection. Front Med. 2020;14(2):185–92. 10.1007/s11684-020-0754-0 32170560 PMC7088738

[9] Aragao DS, Cunha TS, Arita DY, Andrade MC, Fernandes AB, Watanabe IK, et al. Purification and characterization of angiotensin converting enzyme 2 (ACE2) from murine model of mesangial cell in culture. Int J Biol Macromol. 2011;49(1):79–84. 10.1016/j.ijbiomac.2011.03.018 21470562 PMC7112419

[10] Su Z, Zimpelmann J, Burns KD. Angiotensin-(1–7) inhibits angiotensin II-stimulated phosphorylation of MAP kinases in proximal tubular cells. Kidney Int. 2006;69(12):2212–8. 10.1038/sj.ki.5001509 . 16672906

[11] Hamming I, Timens W, Bulthuis ML, Lely AT, Navis G, van Goor H. Tissue distribution of ACE2 protein, the functional receptor for SARS coronavirus. A first step in understanding SARS pathogenesis. J Pathol. 2004;203(2):631–7. 10.1002/path.1570 15141377 PMC7167720

[12] Pan XW, Xu D, Zhang H, Zhou W, Wang LH, Cui XG. Identification of a potential mechanism of acute kidney injury during the COVID-19 outbreak: a study based on single-cell transcriptome analysis. Intensive Care Med. 2020;46(6):1114–6. 10.1007/s00134-020-06026-1 32236644 PMC7106051

[13] Puelles VG, Lutgehetmann M, Lindenmeyer MT, Sperhake JP, Wong MN, Allweiss L, et al. Multiorgan and Renal Tropism of SARS-CoV-2. N Engl J Med. 2020;383(6):590–2. 10.1056/NEJMc2011400 32402155 PMC7240771

[14] Richardson S, Hirsch JS, Narasimhan M, Crawford JM, McGinn T, Davidson KW, et al. Presenting Characteristics, Comorbidities, and Outcomes Among 5700 Patients Hospitalized With COVID-19 in the New York City Area. JAMA. 2020;323(20):2052–9. 10.1001/jama.2020.6775 32320003 PMC7177629

[15] Schurink B, Roos E, Radonic T, Barbe E, Bouman CSC, de Boer HH, et al. Viral presence and immunopathology in patients with lethal COVID-19: a prospective autopsy cohort study. Lancet Microbe. 2020;1(7):e290–e9. 10.1016/S2666-5247(20)30144-0 33015653 PMC7518879

[16] Hanley B, Naresh KN, Roufosse C, Nicholson AG, Weir J, Cooke GS, et al. Histopathological findings and viral tropism in UK patients with severe fatal COVID-19: a post-mortem study. Lancet Microbe. 2020;1(6):e245–e53. 10.1016/S2666-5247(20)30115-4 32844161 PMC7440861

[17] Braun F, Lutgehetmann M, Pfefferle S, Wong MN, Carsten A, Lindenmeyer MT, et al. SARS-CoV-2 renal tropism associates with acute kidney injury. Lancet. 2020;396(10251):597–8. 10.1016/S0140-6736(20)31759-1 32818439 PMC7431179

[18] Kudose S, Batal I, Santoriello D, Xu K, Barasch J, Peleg Y, et al. Kidney Biopsy Findings in Patients with COVID-19. J Am Soc Nephrol. 2020;31(9):1959–68. 10.1681/ASN.2020060802 32680910 PMC7461665

[19] Ronco C, Navalesi P, Vincent JL. Coronavirus epidemic: preparing for extracorporeal organ support in intensive care. Lancet Respir Med. 2020;8(3):240–1. 10.1016/S2213-2600(20)30060-6 32035509 PMC7154507

[20] Fisher M, Neugarten J, Bellin E, Yunes M, Stahl L, Johns TS, et al. AKI in Hospitalized Patients with and without COVID-19: A Comparison Study. J Am Soc Nephrol. 2020;31(9):2145–57. 10.1681/ASN.2020040509 32669322 PMC7461660

[21] Cheng Y, Luo R, Wang K, Zhang M, Wang Z, Dong L, et al. Kidney disease is associated with in-hospital death of patients with COVID-19. Kidney Int. 2020;97(5):829–38. 10.1016/j.kint.2020.03.005 32247631 PMC7110296

[22] Zhou F, Yu T, Du R, Fan G, Liu Y, Liu Z, et al. Clinical course and risk factors for mortality of adult inpatients with COVID-19 in Wuhan, China: a retrospective cohort study. Lancet. 2020;395(10229):1054–62. 10.1016/S0140-6736(20)30566-3 32171076 PMC7270627

[23] Batlle D, Soler MJ, Sparks MA, Hiremath S, South AM, Welling PA, et al. Acute Kidney Injury in COVID-19: Emerging Evidence of a Distinct Pathophysiology. J Am Soc Nephrol. 2020;31(7):1380–3. 10.1681/ASN.2020040419 32366514 PMC7350999

[24] Arroyo-Johnson C, Mincey KD. Obesity Epidemiology Worldwide. Gastroenterol Clin North Am. 2016;45(4):571–9. 10.1016/j.gtc.2016.07.012 27837773 PMC5599163

[25] Khwaja A. KDIGO clinical practice guidelines for acute kidney injury. Nephron Clin Pract. 2012;120(4):c179–84. 10.1159/000339789 . 22890468

[26] Pelletier K, Lafrance JP, Roy L, Charest M, Belanger MC, Cailhier JF, et al. Estimating glomerular filtration rate in patients with acute kidney injury: a prospective multicenter study of diagnostic accuracy. Nephrol Dial Transplant. 2020;35(11):1886–93. 10.1093/ndt/gfz178 . 33151336

[27] Force ADT, Ranieri VM, Rubenfeld GD, Thompson BT, Ferguson ND, Caldwell E, et al. Acute respiratory distress syndrome: the Berlin Definition. JAMA. 2012;307(23):2526–33. 10.1001/jama.2012.5669 . 22797452

[28] Alhazzani W, Moller MH, Arabi YM, Loeb M, Gong MN, Fan E, et al. Surviving Sepsis Campaign: Guidelines on the Management of Critically Ill Adults with Coronavirus Disease 2019 (COVID-19). Crit Care Med. 2020;48(6):e440–e69. 10.1097/CCM.0000000000004363 32224769 PMC7176264

[29] Hirsch JS, Ng JH, Ross DW, Sharma P, Shah HH, Barnett RL, et al. Acute kidney injury in patients hospitalized with COVID-19. Kidney Int. 2020;98(1):209–18. 10.1016/j.kint.2020.05.006 32416116 PMC7229463

[30] Lee JR, Silberzweig J, Akchurin O, Choi ME, Srivatana V, Lin J, et al. Characteristics of Acute Kidney Injury in Hospitalized COVID-19 Patients in an Urban Academic Medical Center. Clin J Am Soc Nephrol. 2020. 10.2215/CJN.07440520 . 32948642 PMC7863636

[31] Hasan I, Rashid T, Suliman S, Amer H, R MC, Mai ML, et al. Predictors of disease severity and outcome of hospitalized renal transplant recipients with COVID-19 infection: A systematic review of a globally representative sample. Rom J Intern Med. 2020. 10.2478/rjim-2020-0034 . 33155999

[32] Zhang J, Li J, Su L, Yang J, Jiang X, Jiang N, et al. [Clinical characteristics and risk factors of acute kidney injury in coronavirus disease 2019]. Zhonghua Wei Zhong Bing Ji Jiu Yi Xue. 2020;32(4):407–11. 10.3760/cma.j.cn121430-20200302-00198 . 32527342

[33] Joseph A, Zafrani L, Mabrouki A, Azoulay E, Darmon M. Acute kidney injury in patients with SARS-CoV-2 infection. Ann Intensive Care. 2020;10(1):117. 10.1186/s13613-020-00734-z 32880774 PMC7471244

[34] Riva G, Nasillo V, Tagliafico E, Trenti T, Comoli P, Luppi M. COVID-19: more than a cytokine storm. Crit Care. 2020;24(1):549. 10.1186/s13054-020-03267-w 32887652 PMC7472946

[35] Lane JCE, Weaver J, Kostka K, Duarte-Salles T, Abrahao MTF, Alghoul H, et al. Risk of hydroxychloroquine alone and in combination with azithromycin in the treatment of rheumatoid arthritis: a multinational, retrospective study. Lancet Rheumatol. 2020;2(11):e698–e711. 10.1016/S2665-9913(20)30276-9 32864627 PMC7442425

[36] Mercuro NJ, Yen CF, Shim DJ, Maher TR, McCoy CM, Zimetbaum PJ, et al. Risk of QT Interval Prolongation Associated With Use of Hydroxychloroquine With or Without Concomitant Azithromycin Among Hospitalized Patients Testing Positive for Coronavirus Disease 2019 (COVID-19). JAMA Cardiol. 2020;5(9):1036–41. 10.1001/jamacardio.2020.1834 32936252 PMC7195692

[37] Gevers S, Kwa MSG, Wijnans E, van Nieuwkoop C. Safety considerations for chloroquine and hydroxychloroquine in the treatment of COVID-19. Clin Microbiol Infect. 2020;26(9):1276–7. 10.1016/j.cmi.2020.05.006 32422406 PMC7228887

[38] Mauthe M, Orhon I, Rocchi C, Zhou X, Luhr M, Hijlkema KJ, et al. Chloroquine inhibits autophagic flux by decreasing autophagosome-lysosome fusion. Autophagy. 2018;14(8):1435–55. 10.1080/15548627.2018.1474314 29940786 PMC6103682

[39] Festa BP, Chen Z, Berquez M, Debaix H, Tokonami N, Prange JA, et al. Impaired autophagy bridges lysosomal storage disease and epithelial dysfunction in the kidney. Nat Commun. 2018;9(1):161. 10.1038/s41467-017-02536-7 29323117 PMC5765140

[40] Wong J, Munsif M, O’Hehir R, Hew M, Dabscheck E. Hypotensive episodes associated with azithromycin infusion: a potentially fatal adverse drug reaction. Respirol Case Rep. 2019;7(7):e00464. 10.1002/rcr2.464 31406576 PMC6682541

[41] Woodruff AE, Meaney CJ, Hansen EA, Prescott GM. Azithromycin-Induced, Biopsy-Proven Acute Interstitial Nephritis in an Adult Successfully Treated with Low-Dose Corticosteroids. Pharmacotherapy. 2015;35(11):e169–74. 10.1002/phar.1660 . 26598102

[42] Edelstein CL, Venkatachalam MA, Dong Z. Autophagy inhibition by chloroquine and hydroxychloroquine could adversely affect acute kidney injury and other organ injury in critically ill patients with COVID-19. Kidney Int. 2020;98(1):234–5. 10.1016/j.kint.2020.05.001 32437765 PMC7207116

[43] Yang X, Yu Y, Xu J, Shu H, Xia J, Liu H, et al. Clinical course and outcomes of critically ill patients with SARS-CoV-2 pneumonia in Wuhan, China: a single-centered, retrospective, observational study. Lancet Respir Med. 2020;8(5):475–81. 10.1016/S2213-2600(20)30079-5 32105632 PMC7102538

[44] Liu J, Liu Y, Xiang P, Pu L, Xiong H, Li C, et al. Neutrophil-to-lymphocyte ratio predicts critical illness patients with 2019 coronavirus disease in the early stage. J Transl Med. 2020;18(1):206. 10.1186/s12967-020-02374-0 32434518 PMC7237880

[45] Chen Q, Zhu S, Liao J, He W. Study of Acute Kidney Injury on 309 Hypertensive Inpatients with ACEI/ARB—Diuretic Treatment. J Natl Med Assoc. 2018;110(3):287–96. 10.1016/j.jnma.2017.06.008 . 29778133

[46] Li H, Liu L, Zhang D, Xu J, Dai H, Tang N, et al. SARS-CoV-2 and viral sepsis: observations and hypotheses. Lancet. 2020;395(10235):1517–20. 10.1016/S0140-6736(20)30920-X 32311318 PMC7164875

[47] Iba T, Levy JH, Connors JM, Warkentin TE, Thachil J, Levi M. The unique characteristics of COVID-19 coagulopathy. Crit Care. 2020;24(1):360. 10.1186/s13054-020-03077-0 32552865 PMC7301352

[48] Russo E, Esposito P, Taramasso L, Magnasco L, Saio M, Briano F, et al. Kidney disease and all-cause mortality in patients with COVID-19 hospitalized in Genoa, Northern Italy. J Nephrol. 2020. 10.1007/s40620-020-00875-1 33025516 PMC7538179

[49] Chou CY, Yeh HC, Chen W, Liu JH, Lin HH, Liu YL, et al. Norepinephrine and hospital mortality in critically ill patients undergoing continuous renal replacement therapy. Artif Organs. 2011;35(2):E11–7. 10.1111/j.1525-1594.2010.01115.x . 21314834

[50] Lankadeva YR, Kosaka J, Evans RG, Bailey SR, Bellomo R, May CN. Intrarenal and urinary oxygenation during norepinephrine resuscitation in ovine septic acute kidney injury. Kidney Int. 2016;90(1):100–8. 10.1016/j.kint.2016.02.017 . 27165831

[51] Tao Z, Xu J, Chen W, Yang Z, Xu X, Liu L, et al. Anemia is associated with severe illness in COVID-19: A retrospective cohort study. J Med Virol. 2021;93(3):1478–88. 10.1002/jmv.26444 32813298 PMC7461220

[52] Scharte M, Fink MP. Red blood cell physiology in critical illness. Crit Care Med. 2003;31(12 Suppl):S651–7. 10.1097/01.CCM.0000098036.90796.ED . 14724462

[53] Weiss G, Ganz T, Goodnough LT. Anemia of inflammation. Blood. 2019;133(1):40–50. 10.1182/blood-2018-06-856500 30401705 PMC6536698

[54] Goes MA, Iizuka IJ, Quinto BM, Dalboni MA, Monte JC, Santos BC, et al. Serum Soluble-Fas, Inflammation, and Anemia in Acute Kidney Injury. Artif Organs. 2018;42(9):E283–E9. 10.1111/aor.12019 . 23566289

[55] Chiloff DM, de Almeida DC, Dalboni MA, Canziani ME, George SK, Morsi AM, et al. Soluble Fas affects erythropoiesis in vitro and acts as a potential predictor of erythropoiesis-stimulating agent therapy in patients with chronic kidney disease. American journal of physiology Renal physiology. 2020;318(4):F861–F9. 10.1152/ajprenal.00433.2019 32003597 PMC7474254

