# Supplementary material for: Acute kidney injury: Incidence, risk factors, and outcomes in severe COVID-19 patients
Source: PLoS One. 2021 May 25;16(5):e0251048. doi: 10.1371/journal.pone.0251048 (PMC8148326; doi:10.1371/journal.pone.0251048)
Supplement: S1 File — (PDF) [file pone.0251048.s001.pdf]

Supplemental Material

Acute Kidney Injury: incidence, risk factors and outcomes in severe COVID-19 patients.

Danilo Candido de Almeida [1¶]; Maria do Carmo Pinho Franco [1¶]; Davi Rettori Pardo dos Santos [1&]; Marina Colella Santos [1&]; Isabela Soucin Maltoni [1&]; Felipe Mascotte [1&]; Alexandra Aparecida de Souza [2&]; Paula Massaroni Pietrobom [3&]; Eduardo Alexandrino Medeiros [3&]; Paulo Roberto Abrão Ferreira [3&]; Flavia Ribeiro Machado [4&]; Miguel Angelo Goes [1&*].

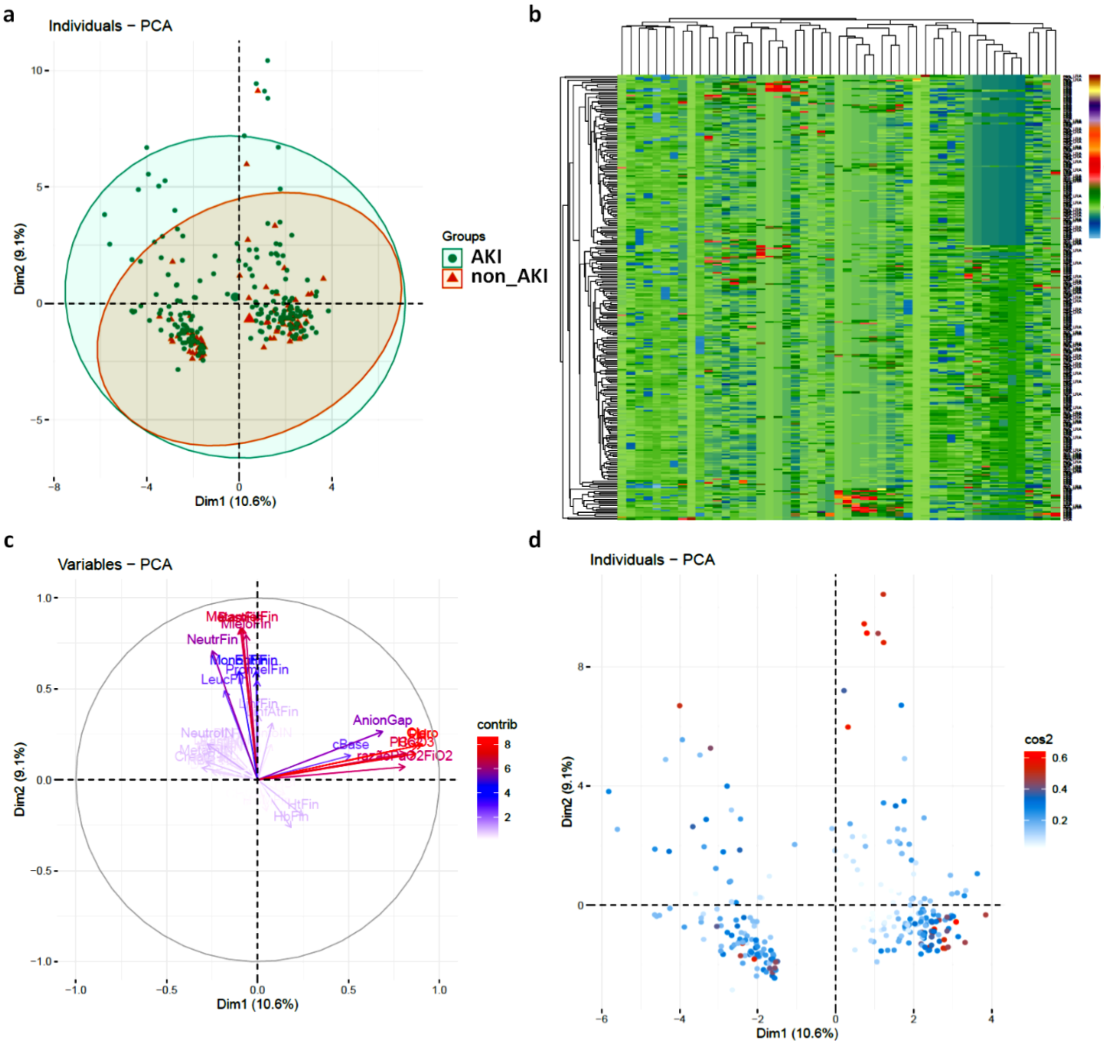

Supplemental Figure 1. Overall multistep classification of covid-19 patients. (**a**) Principal component analysis from numerical variables of all COVID-19 patients allocated at AKI and non-AKI groups; (**b**) clustering heatmap from numerical variables of all COVID-19 patients allocated at AKI and non-AKI groups; (**c**) Vectorial contribution of each numerical variable in principal component analysis; and (**d**) Square cosine indicating the contribution of each component to the squared distance of the observation to the origin. LRA = Cov-AKI and non-LRA = non-AKI. If a component has Higher Cos it is more representative to observation (max:1) (R plots, p<0,05).

Supplemental Methods

The database was generated containing 279 records and 89 variables, which was submitted to a cleaning process eliminating 3 records that contained outliers in the variables: BastIN, BTi and PromielFin. To search important information which could better represents the variables, the database was then submitted to Principal Component Analysis (PCA) in order to reduce the variability in principal components. To obtain the same numerical scale, the analysis was performed using the variables that represent the only "Numerical Discriminative Information" in all database submitted to the transformation process. The PCA was run using the language R and its package FactoMineR with the function PCA parameterized as follows: scale.unit = TRUE, ncp = 2, ind.sup = NULL, quanti.sup = NULL, quali.sup = NULL, row.w = NULL, col.w = NULL, graph = TRUE, axes = c (1,2))

The normalized data were also submitted to cluster analysis. The algorithms directed to this type analysis searched for similar or different aspects among the examples in the database through analyzing of their descriptive attributes and was performed according to their respective grouping distance. Thus, was measured the shorter distance and the greater similarity among data. Using an agglomerative approach, the hierarchical clustering strategy was used to producing a heatmap using the language R and its package stats with the function heatmap parameterized as follows: hclustfun = hclust function (method = "complete", dmeth = "euclidean").

```
To cite R in publications, use:

  R Core Team (2020). R: A language and environment for statistical co
mputing. R Foundation
  for Statistical Computing, Vienna, Austria. URL https://www.R-projec
t.org/.


A BibTeX entry for LaTeX users is

  @Manual{,
    title = {R: A Language and Environment for Statistical Computing},
    author = {{R Core Team}},
    organization = {R Foundation for Statistical Computing},
    address = {Vienna, Austria},
    year = {2020},
    url = {https://www.R-project.org/},
  }


We have invested a lot of time and effort in creating R, please cite i
t when using it for data
analysis. See also 'citation("pkgname")' for citing R packages.
```

```
A BibTeX entry for LaTeX users is

  @Article{,
    title = {{FactoMineR}: A Package for Multivariate Analysis},
    author = {S\'ebastien L\^e and Julie Josse and Fran\c{c}ois Husson
},
    journal = {Journal of Statistical Software},
    year = {2008},
    volume = {25},
    number = {1},
    pages = {1--18},
    doi = {10.18637/jss.v025.i01},
  }


The ‘stats’ package is part of R.  To cite R in publications, use:

  R Core Team (2020). R: A language and environment for statistical co
mputing. R Foundation for
  Statistical Computing, Vienna, Austria. URL https://www.R-project.or
g/.

A BibTeX entry for LaTeX users is

  @Manual{,
    title = {R: A Language and Environment for Statistical Computing},
    author = {{R Core Team}},
    organization = {R Foundation for Statistical Computing},
    address = {Vienna, Austria},
    year = {2020},
    url = {https://www.R-project.org/},
  }


We have invested a lot of time and effort in creating R, please cite i
t when using it for data analysis.
See also ‘citation("pkgname")’ for citing R packages.
```

Supplemental Tables 1. Data of correlation at hospital admission in COVID-19 patients.

	**Admission**														
**non-AKI**	Cr_in	D-dimer_in	Hb_in	HT_in	VCM_in	RDW_in	HCM_in	CHCM_in	Leuc_in	Neutr_in	Eosin_in	Baso_in	Lymph_in	Mono_in	Plate_in
Cr_in	**1.00**														
D-dimer_in	0.08	**1.00**													
Hb_in	-0.03	-0.19	**1.00**												
HT_in	-0.04	-0.17	0.96	**1.00**											
VCM_in	0.08	-0.12	0.05	0.03	**1.00**										
RDW_in	0.15	0.01	-0.09	0.07	-0.14	**1.00**									
HCM_in	-0.05	-0.14	0.11	-0.02	0.87	-0.43	**1.00**								
CHCM_in	0.03	-0.08	0.14	-0.12	0.07	-0.62	0.48	**1.00**							
Leuc_in	-0.01	0.26	0.14	0.18	-0.01	0.18	-0.08	-0.15	**1.00**						
Neutr_in	0.00	0.23	0.14	0.17	-0.05	0.16	-0.10	-0.11	0.98	**1.00**					
Eosin_in	-0.02	0.11	-0.05	0.02	0.07	0.16	-0.05	-0.30	0.06	-0.04	**1.00**				
Baso_in	0.02	-0.07	0.06	0.11	0.26	0.17	0.09	-0.14	-0.04	-0.08	0.32	**1.00**			
Lymph_in	-0.02	0.14	0.04	0.08	0.16	0.10	0.08	-0.14	0.38	0.22	0.45	0.33	**1.00**		
Mono_in	-0.01	0.19	0.10	0.14	0.04	0.10	-0.03	-0.18	0.70	0.64	0.20	-0.09	0.24	**1.00**	
Plate_in	0.04	-0.21	0.16	0.18	0.01	0.11	-0.05	-0.11	0.25	0.22	0.14	0.03	0.25	0.18	**1.00**
	**Admission**														
**Cov-AKI**	Cr_in	D-dimer_in	Hb_in	HT_in	VCM_in	RDW_in	HCM_in	CHCM_in	Leuc_in	Neutr_in	Eosin_in	Baso_in	Lymph_in	Mono_in	Plate_in
Cr_in	**1.00**														
D-dimer_in	0.08	**1.00**													
Hb_in	-0.24	-0.13	**1.00**												
HT_in	-0.24	-0.11	0.94	**1.00**											
VCM_in	-0.05	0.02	-0.07	0.09	**1.00**										
RDW_in	-0.01	0.16	-0.31	-0.33	-0.18	**1.00**									
HCM_in	-0.03	0.01	0.05	0.15	0.93	-0.33	**1.00**								
CHCM_in	0.07	-0.01	0.27	0.19	-0.02	-0.43	0.34	**1.00**							
Leuc_in	0.03	0.16	-0.07	-0.05	0.03	0.13	-0.02	-0.12	**1.00**						
Neutr_in	0.15	0.12	-0.11	-0.09	0.00	0.16	-0.06	-0.15	0.53	**1.00**					
Eosin_in	0.01	-0.06	0.06	-0.21	-0.36	0.23	-0.38	-0.18	0.01	-0.12	**1.00**				
Baso_in	0.02	0.14	-0.10	-0.09	0.13	0.10	0.14	0.05	0.17	0.09	0.00	**1.00**			
Lymph_in	-0.12	0.00	-0.04	0.02	0.00	0.11	-0.07	-0.17	0.13	0.00	-0.03	0.16	**1.00**		
Mono_in	0.08	0.04	-0.11	-0.08	-0.04	0.13	-0.13	-0.20	0.33	0.43	0.10	0.04	0.27	**1.00**	
Plate_in	-0.02	0.16	-0.18	-0.11	-0.04	0.01	-0.06	-0.05	0.31	0.38	-0.15	0.12	0.24	0.35	**1.00**

Supplemental Tables 2. Data of correlation at hospital discharge in COVID-19 patients.

	Discharge														
**non-AKI**	Cr_out	D-dimer_out	Hb_out	HT_out	VCM_out	RDW_out	HCM_out	CHCM_out	Leuc_out	Neutr_out	Eosout_out	Baso_out	Lymph_out	Mono_out	Plate_out
Cr_out	**1.00**														
D-dimer_out	-0.09	**1.00**													
Hb_out	-0.26	-0.08	**1.00**												
HT_out	-0.30	-0.01	0.98	**1.00**											
VCM_out	0.09	-0.07	0.04	0.00	**1.00**										
RDW_out	-0.06	0.29	-0.48	-0.40	-0.16	**1.00**									
HCM_out	0.10	-0.17	0.12	0.03	0.74	-0.33	**1.00**								
CHCM_out	0.13	-0.36	0.31	0.14	0.21	-0.54	0.53	**1.00**							
Leuc_out	0.56	-0.06	-0.30	-0.33	0.04	0.01	0.09	0.14	**1.00**						
Neutr_out	0.48	-0.05	-0.32	-0.36	0.05	0.03	0.09	0.16	0.97	**1.00**					
Eosout_out	-0.02	-0.06	0.05	0.06	0.05	0.09	0.06	-0.03	0.12	0.02	**1.00**				
Baso_out	-0.04	0.20	0.08	0.07	0.08	0.04	-0.01	0.04	0.19	0.20	0.39	**1.00**			
Lymph_out	0.25	-0.11	-0.10	-0.06	-0.11	0.16	-0.24	-0.20	0.04	-0.04	0.14	-0.03	**1.00**		
Mono_out	0.20	-0.10	-0.05	-0.08	-0.01	-0.13	-0.02	0.17	0.58	0.53	0.06	0.30	0.02	**1.00**	
Plate_out	-0.07	-0.17	0.12	0.13	-0.36	-0.27	-0.26	-0.01	0.12	0.10	-0.14	-0.04	-0.03	0.19	**1.00**
	**Discharge**														
**Cov-AKI**	Cr_out	D-dimer_out	Hb_out	HT_out	VCM_out	RDW_out	HCM_out	CHCM_out	Leuc_out	Neutr_out	Eosout_out	Baso_out	Lymph_out	Mono_out	Plate_out
Cr_out	**1.00**														
D-dimer_out	-0.01	**1.00**													
Hb_out	-0.14	-0.28	**1.00**												
HT_out	-0.10	-0.24	0.93	**1.00**											
VCM_out	0.02	0.02	-0.22	0.05	**1.00**										
RDW_out	0.08	0.01	-0.42	-0.40	-0.58	**1.00**									
HCM_out	-0.09	-0.21	0.03	0.14	0.73	-0.57	**1.00**								
CHCM_out	-0.16	-0.05	0.36	0.24	-0.13	-0.27	0.22	**1.00**							
Leuc_out	0.19	0.32	-0.39	-0.37	0.08	0.33	-0.20	-0.24	**1.00**						
Neutr_out	0.22	0.30	-0.40	-0.37	0.06	0.13	-0.17	-0.23	0.97	**1.00**					
Eosout_out	0.00	-0.07	-0.15	-0.34	-0.68	0.96	-0.64	-0.24	0.28	0.05	**1.00**				
Baso_out	-0.07	-0.11	-0.13	-0.09	0.03	-0.10	0.06	-0.06	-0.09	-0.13	-0.06	**1.00**			
Lymph_out	-0.20	-0.02	0.07	0.07	-0.05	-0.05	0.01	-0.06	-0.01	-0.17	0.10	0.03	**1.00**		
Mono_out	-0.06	0.34	-0.04	0.02	0.10	-0.12	-0.14	-0.02	0.55	0.44	-0.20	0.07	0.15	**1.00**	
Plate_out	-0.22	-0.14	0.28	0.31	0.00	-0.25	0.05	0.10	-0.06	-0.11	-0.24	0.18	0.26	0.15	**1.00**
